# Automated Analysis and Classification of Histological Tissue Features by Multi-Dimensional Microscopic Molecular Profiling

**DOI:** 10.1371/journal.pone.0128975

**Published:** 2015-07-15

**Authors:** Daniel P. Riordan, Sushama Varma, Robert B. West, Patrick O. Brown

**Affiliations:** 1 Department of Biochemistry, Stanford University School of Medicine, Stanford, California, United States of America; 2 Howard Hughes Medical Institute, Stanford University School of Medicine, Stanford, California, United States of America; 3 Department of Pathology, Stanford University School of Medicine, Stanford, California, United States of America; Universidad Carlos III of Madrid, SPAIN

## Abstract

Characterization of the molecular attributes and spatial arrangements of cells and features within complex human tissues provides a critical basis for understanding processes involved in development and disease. Moreover, the ability to automate steps in the analysis and interpretation of histological images that currently require manual inspection by pathologists could revolutionize medical diagnostics. Toward this end, we developed a new imaging approach called multidimensional microscopic molecular profiling (MMMP) that can measure several independent molecular properties *in situ* at subcellular resolution for the same tissue specimen. MMMP involves repeated cycles of antibody or histochemical staining, imaging, and signal removal, which ultimately can generate information analogous to a multidimensional flow cytometry analysis on intact tissue sections. We performed a MMMP analysis on a tissue microarray containing a diverse set of 102 human tissues using a panel of 15 informative antibody and 5 histochemical stains plus DAPI. Large-scale unsupervised analysis of MMMP data, and visualization of the resulting classifications, identified molecular profiles that were associated with functional tissue features. We then directly annotated H&E images from this MMMP series such that canonical histological features of interest (e.g. blood vessels, epithelium, red blood cells) were individually labeled. By integrating image annotation data, we identified molecular signatures that were associated with specific histological annotations and we developed statistical models for automatically classifying these features. The classification accuracy for automated histology labeling was objectively evaluated using a cross-validation strategy, and significant accuracy (with a median per-pixel rate of 77% per feature from 15 annotated samples) for *de novo* feature prediction was obtained. These results suggest that high-dimensional profiling may advance the development of computer-based systems for automatically parsing relevant histological and cellular features from molecular imaging data of arbitrary human tissue samples, and can provide a framework and resource to spur the optimization of these technologies.

## Introduction

Microscopic examination of cellular morphology and structure is a classical approach that has provided an invaluable foundation for analyzing the function, development, and organization of complex tissues. Accordingly, a large number of biomedical research and diagnostic methods are based on the identification of architectural tissue features by histopathology [1–3]. At the same time, highly multiplexed interrogation of the molecular components of different samples has proven to be a tremendously rich complementary strategy for their characterization and classification. Large-scale molecular studies based on microarray analysis, high-throughput sequencing, and proteomic approaches have clearly demonstrated the advantages of quantitative multi-dimensional profiling for identifying functionally important subtypes of cancers and other cellular states with important clinical ramifications [4–6]. Nevertheless, these techniques often require physical disruption of the interrogated samples, which sacrifices critical spatial information related to the individual cells and their native positional arrangements and relationships within intact specimens. Therefore, technologies that enable the acquisition of high-dimensional molecular profiles while retaining the spatial integrity of the examined material offer great potential for advancing the detailed characterization of important biological samples.

Accordingly, several different approaches for multiplex *in situ* profiling of tissue sections have been pursued. Traditional multi-color fluorescence microscopy enables the simultaneous monitoring of up to five spectrally resolvable dyes at once using standard optical filters, and up to seven fluorophores may be detected with multispectral approaches [7]. In order to overcome these limitations, several independent strategies based on serial staining and imaging have been developed which greatly expand the number of molecular characteristics that can be assayed from an individual sample [8–13]. Other novel strategies based on mass spectrometry imaging modalities for the simultaneous detection of up to 32 distinct markers have also been successfully applied to the dissection of cellular states from intact clinically relevant tissue samples, further demonstrating the exceptional power of such multiplex technologies in combination with advanced analytical techniques [14–18].

Here we describe a new approach, called multi-dimensional microscopic molecular profiling (MMMP), that can measure several independent molecular properties *in situ* at subcellular resolution for the same tissue specimen. The MMMP procedure was adapted to work with formalin-fixed paraffin-embedded tissue samples that are commonly used for clinical specimens, and therefore was compatible with the use of tissue microarray (TMA) slides [19]. We conducted a MMMP analysis of a TMA containing 102 unique human tissue sections, and performed several large-scale data analyses on the resulting multi-dimensional datasets associated with each sample, which we report here. By integrating manual annotation of relevant cellular and histological properties with our molecular imaging data, we further exploited these data to address the problem of automated histological feature recognition within tissue sections.

## Results

### Overview of Multi-dimensional Microscopic Molecular Profiling (MMMP)

**Fig 1:**
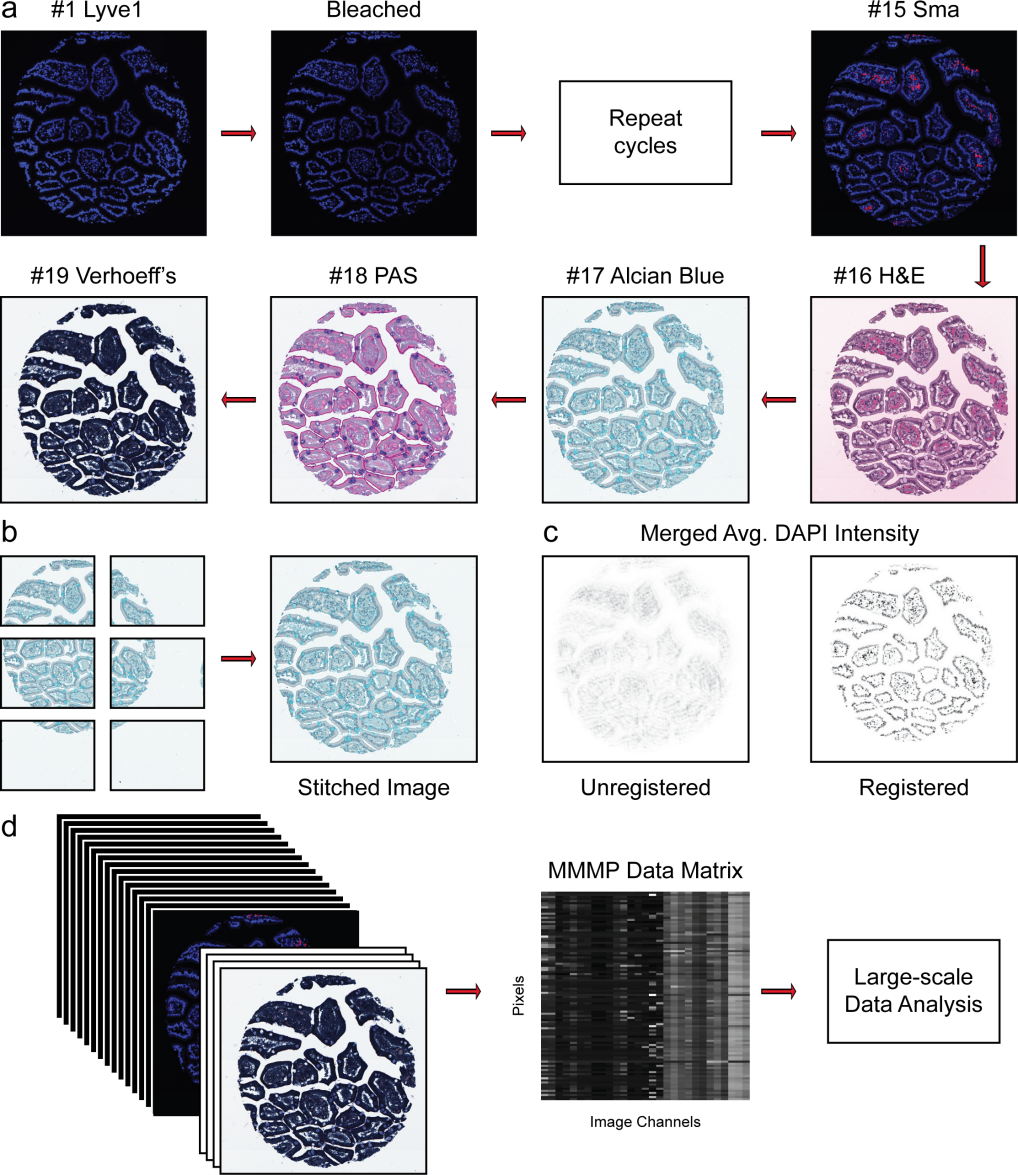
Overview of Multi-dimensional Microscopic Molecular Profiling (MMMP). The overall MMMP approach is depicted using an example tissue section from normal human duodenum (sample #1.9.7). (a) Slides were subjected to repeated cycles of staining and imaging with fluorescent primary antibodies and DAPI. At the end of each cycle, fluorescent signal was removed by a chemical bleaching process, and slides were again imaged, before proceeding to the next round of this iterative procedure. After the final antibody stain (#15 Sma), slides were analyzed with a series of histochemical stains. (b) A set of tiling images spanning each tissue section was initially generated by the microscope system. The tiling images were then computationally ‘stitched’ together to produce a single image per staining cycle for each sample. (c) Image registration was performed to align images from the same tissue section across cycles. Mean intensities of the DAPI signal from all immuno-fluorescence images are shown from before (Unregistered) and after (Registered) the image registration procedure was completed. (d) Following registration, signal intensities from the relevant channels for each image (columns) in the MMMP series were extracted for each pixel (rows) within the tissue section and compiled into a large data matrix of *in situ* molecular profiles.

In order to simultaneously analyze several molecular properties of cells while preserving their native spatial arrangements in tissues, we developed an imaging approach called multi-dimensional microscopic molecular profiling (MMMP) (Fig 1). The MMMP strategy involves iterative cycles of antibody (or histochemical) staining, imaging, and signal removal, which can ultimately generate information analogous to a multi-parameter flow cytometry analysis for intact tissue sections. MMMP is conceptually similar to other serial staining techniques with greater efficiency that have been used for multiplex profiling of biological samples [10–13]. We also adapted MMMP to be compatible with formalin-fixed paraffin embedded (FFPE) samples contained on a tissue microarray (TMA), enabling us to simultaneously analyze on the order of hundreds of distinct tissue sections in parallel. The overall approach is illustrated schematically for a single example of a human duodenum tissue sample in Fig 1. Tissue sections were subjected to multiple rounds of imaging and staining with a panel of fluorescent antibodies and histochemical stains. This process results in a collection of separate images for every tissue section present on the TMA, where each image reports on a distinct molecular aspect of the interrogated samples. As an additional control, we repeated the antibody staining procedure for angiotensin I converting enzyme (Ace) on an independent tissue section generated from the same TMA to ensure that the MMMP cycling procedure did not interfere with successful antibody staining (S2 Fig). As expected, the observed pattern of staining for Ace (which was the fourth antibody applied during the MMMP series) displayed characteristic positivity in sections of human endometrium, fallopian tube, and kidney medulla tissue that was consistent between experiments, suggesting that faithful staining can be achieved through the iterative staining procedure (S2 Fig) [20].

Before extracting multi-dimensional vectors reflecting the joint distribution of staining intensities from each MMMP series, a number of image analysis steps must be performed to properly process and track the thousands of image files that can be generated from a single MMMP experiment. To this end, we established a computational pipeline that automatically facilitates image processing and data extraction for MMMP images [21–23]. The Ariol microscope system (v3.2 Genetix) that we used for imaging captures several tiling images that span each tissue section. We found that the default Ariol software often incorrectly stitched together the tiling images, leading to the erroneous duplication or deletion of regions in the resulting output images that rendered them incompatible with further analysis (S1 Fig). Thus, a necessary first step was to accurately assemble the separate tiling images to produce a single combined image for each tissue section per staining cycle (Fig 1B). We therefore included an image stitching procedure in our computational pipeline that successfully performs this task [22]. The next essential step was to register the stitched images from each cycle such that the pixels corresponding to the same location within each tissue section can be identified and related across all cycles. Our pipeline achieves image registration by using the DAPI fluorescence included in each immunofluorescence image as a fiducial signal to help generate a multi-image alignment for the entire MMMP series (Fig 1C). After image registration, data vectors representing the signal intensities for each stain applied in the image series were extracted for each pixel within the tissue section, and compiled into a MMMP data matrix (Fig 1D). The resulting MMMP matrix of *in situ* molecular profiles can then be investigated and visualized using a variety of large-scale data analysis techniques. The full set of MMMP images generated are available online at the Stanford Tissue Microarray Database [24].

### Unsupervised classification of data from MMMP Analysis of human tissue microarrays

We carried out a MMMP analysis of a human tissue microarray (TMA) containing a diverse set of 102 samples. The adoption of TMAs not only allowed us to simultaneously analyze a large number of independent tissue sections in parallel, but also ensured that all samples were processed under identical experimental conditions. This MMMP study involved a panel of 15 informative primary antibodies (Lyve1, Sparc, Cd34, Ace, Mmp11, Fzd7, Cd105, Col4a2, Ctgf, β-cat, Dkk3, Cd44, Desmin, Col1, and Sma) as well as 5 histochemical stains (Hematoxylin, Eosin, Alcian blue, Periodic Acid-Schiff, and Verhoeff’s stain) plus DAPI. A technical failure of the automated microscope software to properly track and image 28 of the 146 original samples across all of the cycles led to their exclusion from this analysis (S1 Table). Nevertheless, of the 118 samples on the TMA that yielded images for every MMMP cycle, our image processing pipeline was able to successfully generate MMMP matrices for 102 of them (86%). The image channels represented in each dataset included Cy5 antibody staining intensities for all 15 IF images in the series, plus DAPI signals from the first and last IF images, as well as intensities from all three color channels (red, green, and blue) for each of the 4 bright-field images of histochemical stains, yielding a total of 29 dimensions (29 = 15 + 2 + 12) as columns in each MMMP data matrix (Fig 1D).

**Fig 2:**
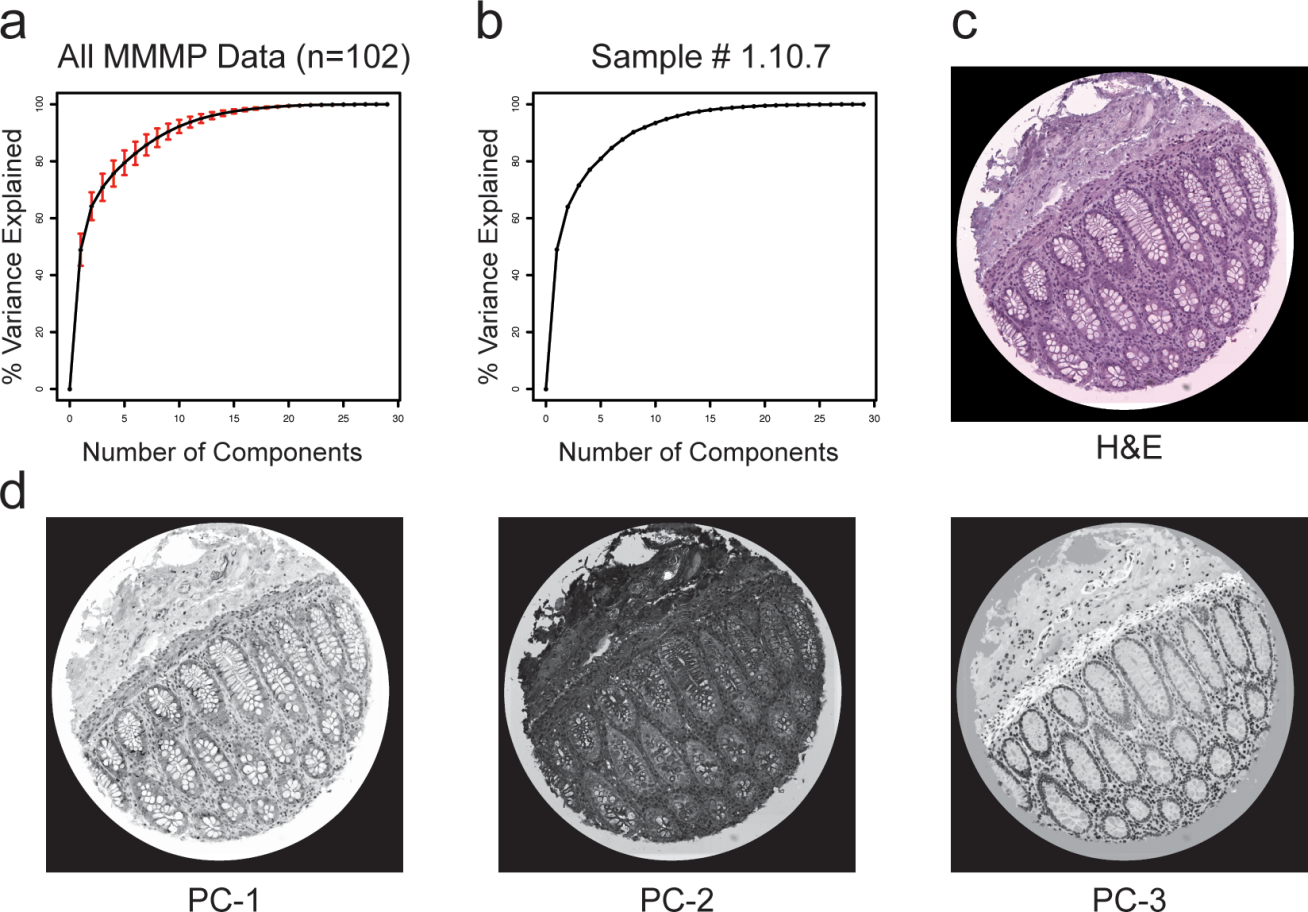
Principal Components Analysis of MMMP Data. (a) The mean value of the cumulative percentage of the variance in the data explained is plotted as a function of the number of principal components for data representing 102 human tissue samples. Red error bars indicate +/–one standard deviation. (b) The cumulative percentage of variance explained by the first *X* principal components is shown as a function of *X* for an example tissue section from the terminal ileum of the small intestine (sample #1.10.7). (c) H&E (hematoxylin and eosin) stained image of sample #1.10.7. (d) Images depicting scaled intensity values for the first three principal components (PC-1, PC-2, and PC-3) of the MMMP data matrix for sample #1.10.7 are shown.

To systematically explore the landscape of molecular profiles present in each tissue section, we performed a number of unsupervised data analyses on the large-scale MMMP datasets generated for each sample. First, we applied Principal Component Analysis (PCA) to our MMMP matrices, as a way of transforming the data along orthogonal axes that capture the variation present in multi-dimensional space (Fig 2). PCA was performed separately on the MMMP dataset obtained for each independent tissue section in order to capture properties of the data that were specific to each individual sample. Summary statistics from the PCA results for all 102 human tissue samples indicate that 20 independent dimensions (components) were typically required to account for at least 99% of the existing variance from the MMMP data, reflecting a high degree of multi-dimensionality observed in the underlying molecular profiles (Fig 2A). PCA results for an example tissue section from the terminal ileum of the small intestine (sample #1.10.7) illustrate how the intensity values for individual principal components can be scaled and visualized as new image files, providing a useful way to facilitate the discovery and interpretation of general trends that emerge from the *in situ* molecular profiles (Fig 2B–2D). In this example, regions of high intensity values for the first principal component (PC-1), which accounts for 49% of the total variation, tend to coincide with the overall presence of nuclear and cytoplasmic tissue staining (Fig 2D, left panel). The second component (PC-2), which explains 15% of the variance, instead appears to be preferentially associated with regions containing extracellular matrix and is anti-correlated with nuclei (Fig 2D, center panel). Finally, the third component (PC-3) accounts for 7.5% of the variance and tends to be positively enriched in nuclei while being selectively decreased in regions containing the *muscularis mucosae* and endothelial cells (Fig 2D, right panel). These results underscore the potential utility for unbiased analysis of deep molecular profiles as a general strategy for identifying specific features with histological relevance within tissue sections.

**Fig 3:**
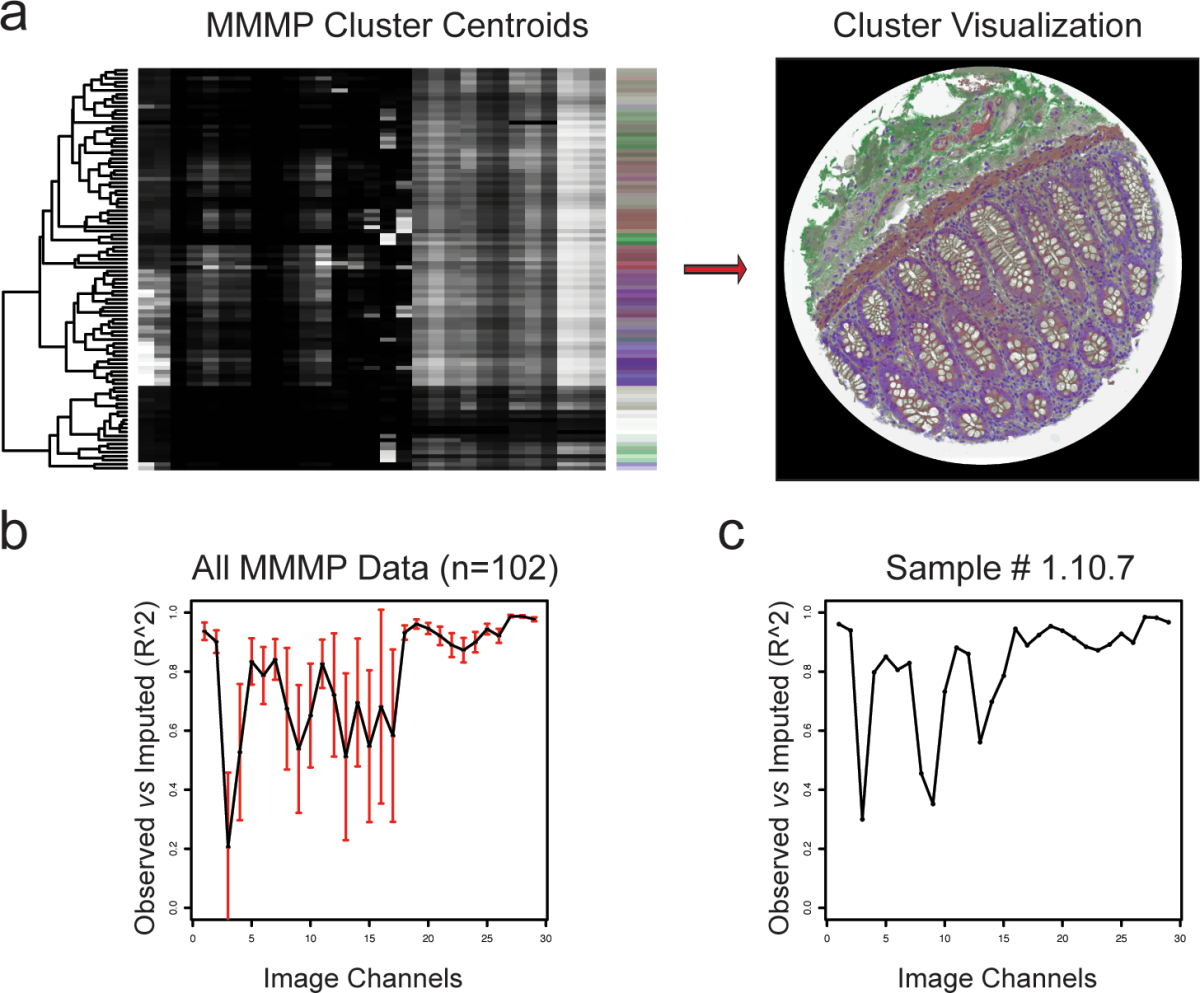
*k*-means Clustering Analysis of MMMP Data. (a) Molecular profiling data from a human small bowel terminal ileum tissue section (#1.10.7) was clustered using a *k*-means algorithm with *k* = 100. The centroid vectors from the *k* clusters were themselves clustered and displayed as rows in the heatmap, where high intensity values are shaded in white and low values in black. Each cluster was designated to the color shown on the right of the heatmap, such that molecularly similar vectors were assigned to visually similar colors. Clustering results were visualized by generating an image where the cluster membership of each pixel is illustrated using the similarity-based color code. (b) Mean values for the squared Pearson’s correlation coefficient (R^2^) over all pixels between their original intensities in the MMMP data and the values imputed based on their cluster membership are plotted for each image channel (data represents 102 tissue samples). Red error bars indicate the standard deviations. (c) R^2^ values for the tissue section presented in (a) (sample #1.10.7) are plotted as in (b).

To further characterize the diversity of molecular profiles observed within each tissue section, we next carried out clustering analysis on the MMMP data from each sample. Using a *k*-means clustering algorithm, we classified each pixel from the MMMP matrix as belonging to one of several (*k* = 100) groups based on the similarity of their molecular profiles (Fig 3). The vector of centroid values for each cluster characterizes the molecular profiles of the pixels that belong to that group, offering a way to identify important differences in staining intensity that were associated with each cluster. A value of *k* designating 100 clusters was arbitrarily chosen in order to simplify the dimensionality while accommodating the possibility for a large range of molecular heterogeneity within each sample, based on testing a range of potential cluster sizes (S3 Fig). In order to effectively visualize the results of clustering analysis involving 100 discrete groups (or conceivably more, for larger values of *k*), we devised a general strategy for color-coding the cluster membership of each pixel such that clusters with similar molecular profiles were assigned similar color values. To accomplish this, we used Kruskal’s non-metric multi-dimensional scaling procedure (implemented in the R function isoMDS) to transform the vectors of cluster centroids into a three-dimensional coordinate system compatible with RGB (red / green / blue) color space (S1 File). The goal of this transformation is to maximize the agreement between the original molecular similarity of clusters and the relatedness of their assigned colors (Fig 3A). After the similarity-based color mapping function has been established, the results from cluster analysis can be readily visualized by generating an image to display the cluster membership of each pixel (Fig 3A). This approach for unsupervised classification and visualization of recurrent patterns derived from the molecular profiles of each tissue section can further enable the identification of significant subcellular or histological features present in each sample. Moreover, it was possible to assess the degree to which the clustering results faithfully captured the full extent of variation in the overall MMMP dataset for each sample. In order to do this, we calculated the agreement between the original intensities of MMMP data from each channel and the “imputed” values that would be obtained if every pixel was assigned a new intensity in each channel determined by the centroid of the cluster to which it belongs. By computing the square of the Pearson correlation coefficient (r^2^), we objectively assessed the extent to which the “imputed” cluster-derived values were a good approximation for the original MMMP data (Fig 3B). The r^2^ values associated with *k*-means clustering of all 102 human tissue samples indicate that most of the variation in molecular profiles is represented in the results of these clustering analyses, with an overall mean r^2^ of 78% across all channels (Fig 3B). The degree of agreement in each channel for the example tissue section of human terminal ileum is shown in Fig 3C.

**Fig 4:**
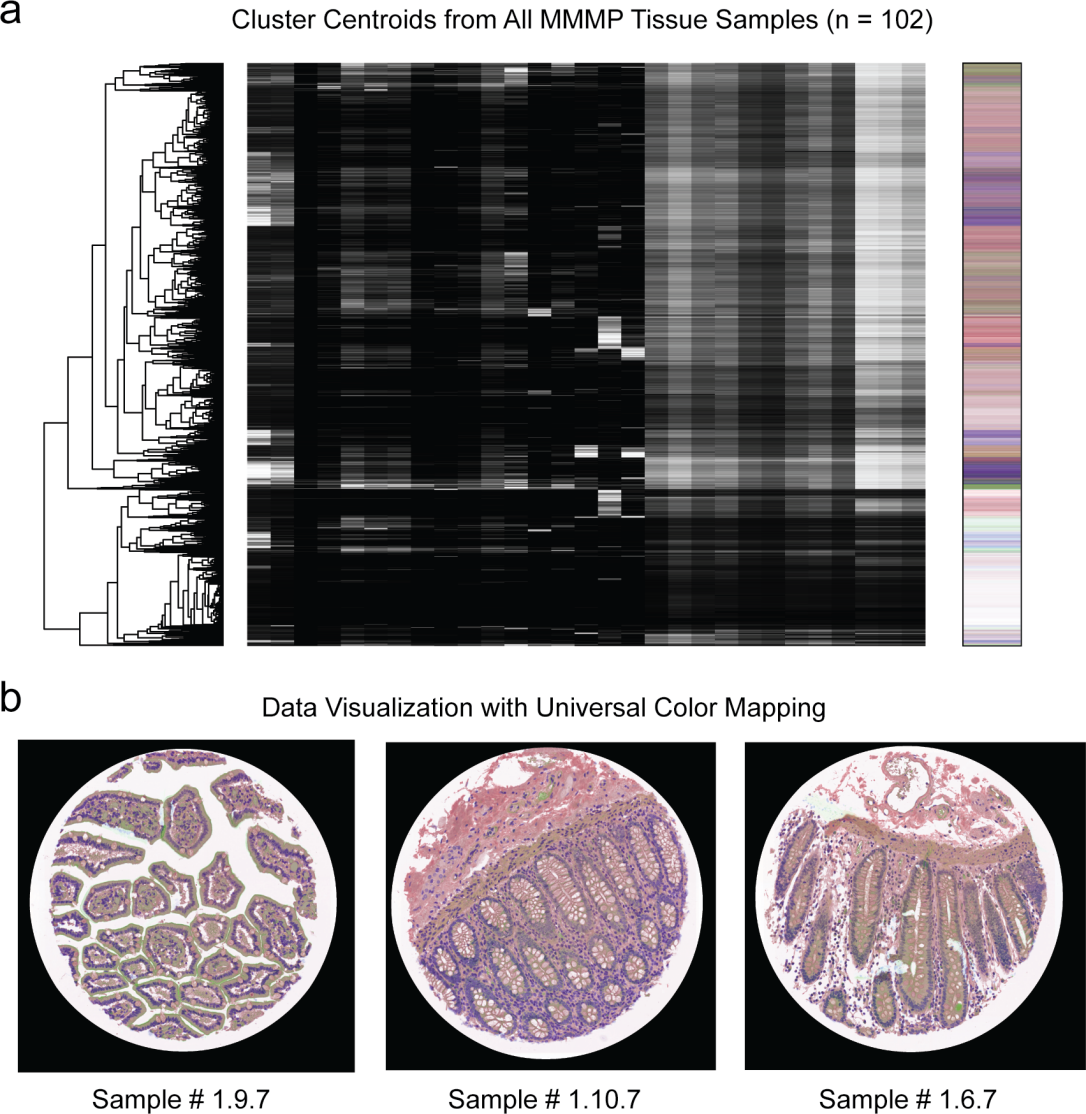
Analysis of Molecular Profiles from Full MMMP Dataset. (a) Cluster centroid vectors from *k*-means clustering of 102 human tissue samples were compiled into a single MMMP data matrix, which was then hierarchically clustered and displayed in heatmap format. Multi-dimensional scaling was used to generate a universal color palette (shown at right) for representing cluster membership based on similarity of molecular profiles. (b) Color-based visualization of the molecular profiles within each of three tissue sections is shown using the universal color mapping function depicted above. Tissue sections were derived from normal human duodenum (#1.9.7), terminal ileum of the small bowel (#1.10.7), and colon (#1.6.7), respectively.

Building on the clustering results from each individual MMMP tissue section, we extended our analysis to examine the full diversity of molecular profiles observed across all of the diverse tissue samples. We therefore extracted the full set of centroid vectors from all clusters identified in the entire dataset, which were then collated into a single MMMP data matrix with 10,200 rows (102 samples x 100 clusters/sample). We then hierarchically clustered all of the vectors, revealing the diversity of trends present in the high-dimensional molecular space occupied by these profiles (Fig 4). We also applied the same similarity-based color mapping procedure as before to generate a single color-coding palette reflecting the overall molecular diversity (Fig 4). This allowed us to visualize each individual MMMP sample using a universal color-coding transformation that can be applied similarly to all of the samples, as shown for the tissue sections of normal human duodenum, terminal ileum, and colon tissue. While this approach may be helpful for facilitating visual comparisons between tissues by imposing an equivalent universal color-mapping scheme onto all samples, it may tend to accentuate more general molecular features that are common across many samples (e.g. nuclei) while potentially obscuring rarer tissue-specific profiles.

### High-dimensional molecular analysis of histological tissue features

The results from our unsupervised analysis and classification of MMMP data suggested that the high-dimensional information-rich molecular profiles that we measured may be useful for enabling automatic identification of important types of cells and tissues in their native microenvironment. In order to pursue this hypothesis more directly, we next integrated manual “expert”-based annotation of histological features with the molecular imaging data from our MMMP analysis.

**Fig 5:**
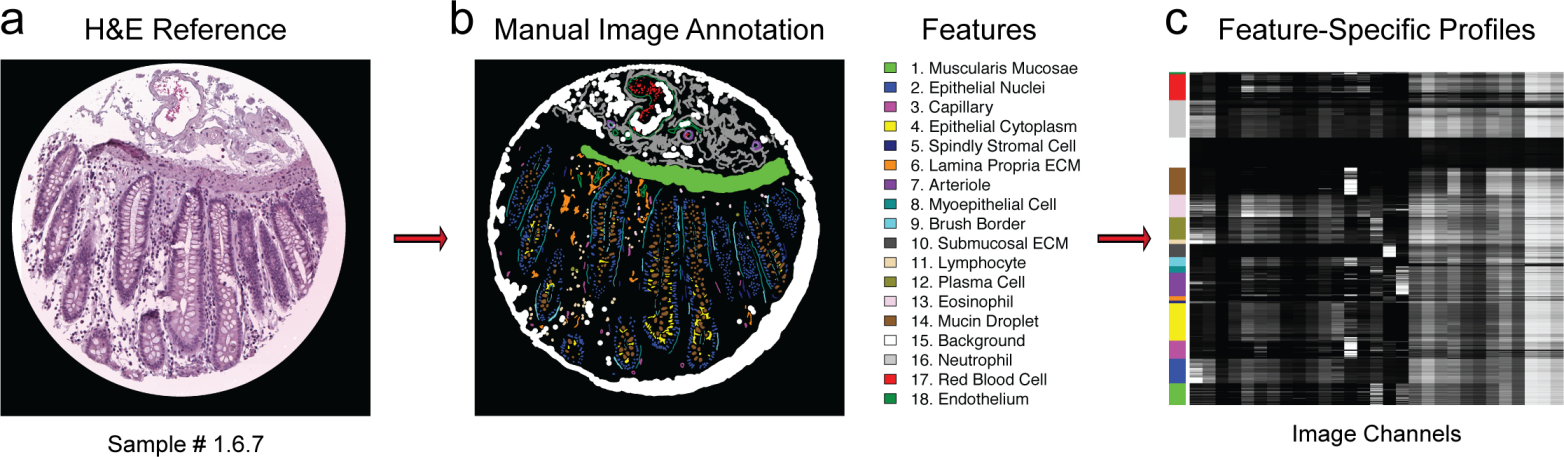
Molecular Analysis of Annotated Histological Features. (a) Hematoxylin and eosin image of a normal human colon tissue section (Sample #1.6.7) was used as a reference for manual annotation of relevant cellular and histological features. (b) Different types of histological features were annotated and assigned arbitrary color codes for visualization purposes. Each labeled feature is displayed using the indicated color scheme. (c) Feature-specific molecular profiles were identified by cluster analysis of MMMP data associated with each annotated histological category, and presented in heatmap format. The associated feature types are indicated by the appropriate color code on the left of the heatmap, following the color scheme used in panel (b).

We manually curated 15 of the tissue sections from our MMMP dataset, using the H&E images as a reference, and focused on annotating well-established histological features from normal human tissues. An example of this approach is illustrated for a sample of normal human colon tissue (Fig 5). We then identified feature-specific molecular signatures that were associated with each histological annotation by analyzing the underlying MMMP vectors that were labeled as belonging to each annotated category (Fig 5C). This strategy enabled us to characterize the molecular diversity of cells and features associated with different histological categories, and the results often recapitulated established relationships that were expected based on known molecular identities (i.e. Collagen 1 staining was enriched in regions of extracellular matrix, Desmin was enriched in the muscularis mucosae, etc.) (Fig 5C).

### Automated recognition of histological tissue features

**Fig 6:**
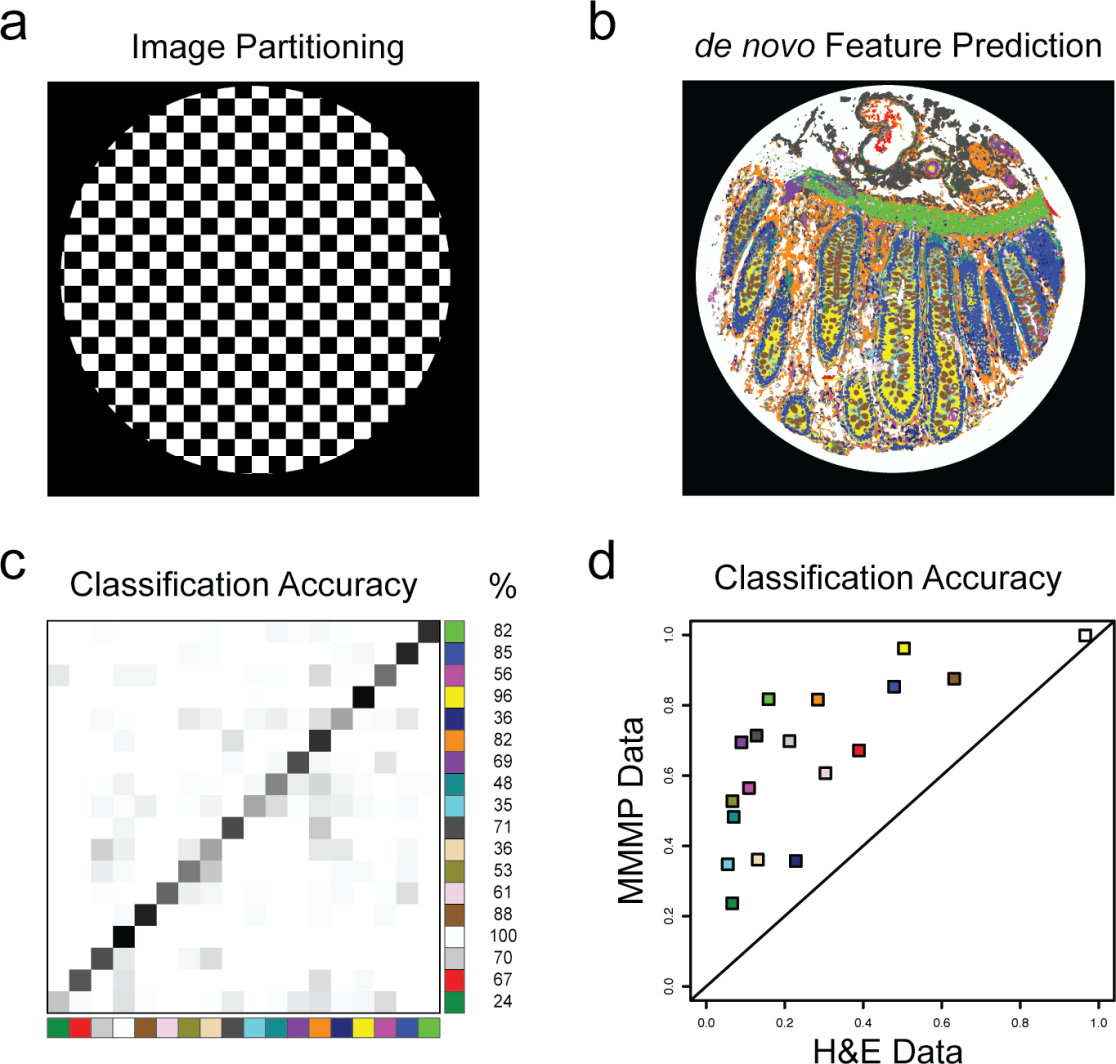
Automated Histology by Classification of MMMP Data. (a) The normal human colon tissue section (Sample #1.6.7) from Fig 5 was partitioned into two independent regions according to a checkerboard pattern (Region 1 = white squares, Region 2 = black squares). Region 1 was used as a “training set” to build a classification model for recognizing histological features based on their molecular profiles, and this model was applied to generate predictions based on data from the “test set” of Region 2. A reciprocal analysis was also performed (i.e. Region 2 = training set, Region 1 = test set) to generate automated labeling of histological categories for the entire tissue section. (b) The results from the automated histology classification are illustrated, using the appropriate color scheme for features as in Fig 5. (c) The accuracy of automated feature recognition was evaluated by comparison to the original annotations. The ‘confusion matrix’ representing the frequency with which pixels manually annotated to each feature (rows) were automatically classified as belonging to any given feature (columns) is shown. The classification accuracy for each feature is shown to the right (overall median accuracy was 68%). (d) The per-feature accuracy for classification based all dimensions of the MMMP data for Sample #1.6.7 is plotted (*y*-axis) versus the corresponding classification accuracy obtained when only data from the hematoxylin and eosin channels was used (*x*-axis).

**Fig 7:**
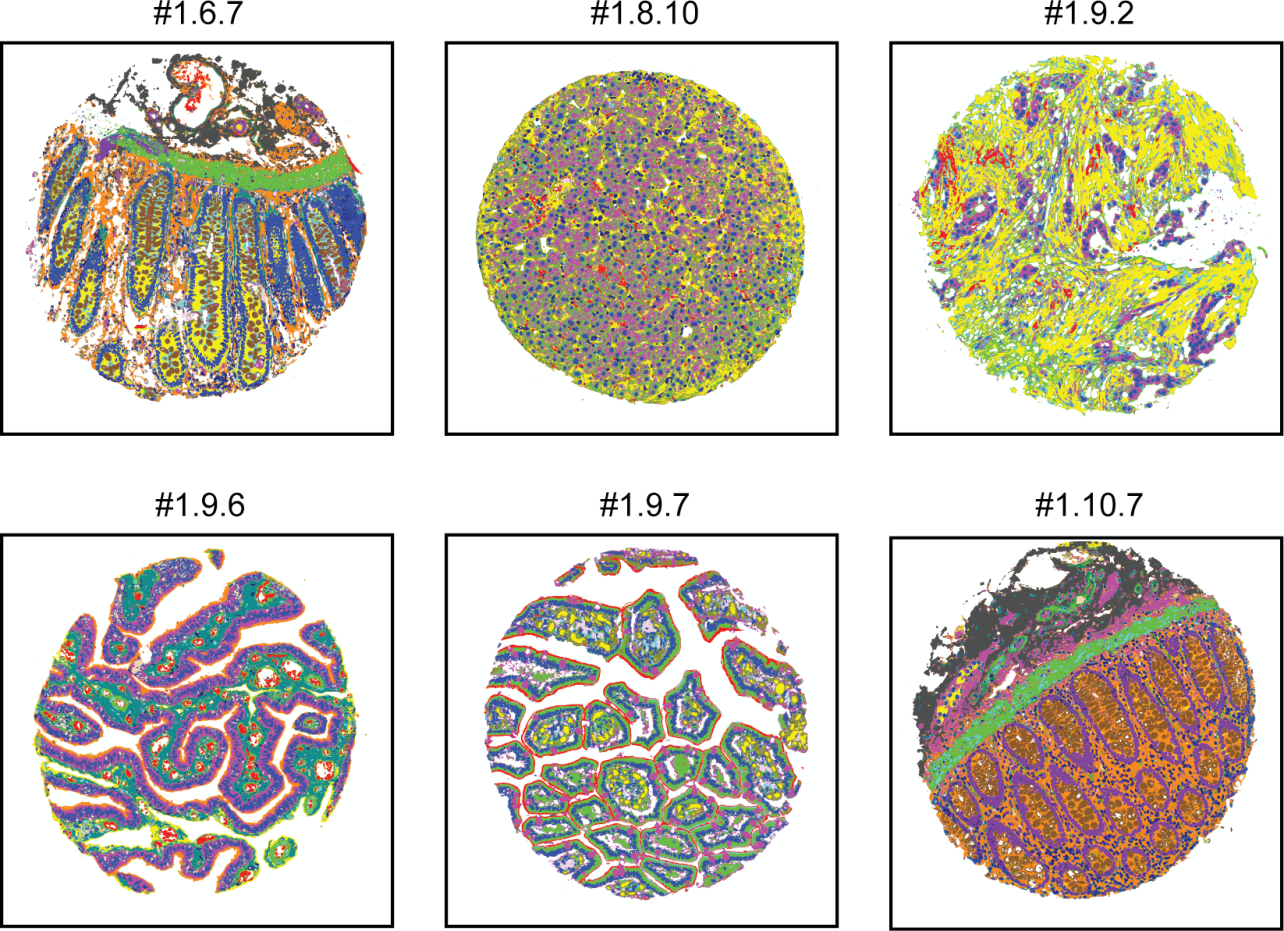
Gallery of Tissue Feature Predictions by Automated Histology. Color-coded output of *de novo* histological feature identification (performed as in Fig 6) is presented for six tissue sections. The average per-pixel classification accuracy across all annotated features for these samples was 72% (median = 75%). Tissue sections displayed are normal human colon (#1.6.7), normal human liver (#1.8.10), human breast with malignant carcinoma (#1.9.2), normal human fallopian tube (#1.9.6), normal human duodenum (#1.9.7), and normal terminal ileum of the small bowel (#1.10.7).

We next set out to develop an automated system for recognizing and predicting individual histological features based on the composition of their molecular profiles, which is demonstrated again using the human colon tissue section as an example (Fig 6). To achieve this, we implemented a cross-validation strategy based on conceptual partitioning of each annotated tissue section into discrete regions that can serve as separate “training” or “test” sets (Fig 6A). After subdividing each image into two regions, the MMMP data vectors associated with Region 1 were used as a “training set” to build a multi-label classifier based on linear discriminant analysis (LDA), which was then applied to data from Region 2 as an independent “test set” to generate automatically predicted feature annotations based on the underlying molecular profiles. Then, the reciprocal procedure was performed, and the automated classifications resulting from both analyses were merged to create a color-coded output image representing the *de novo* prediction of annotated features within the tissue section (Fig 6B). The accuracy of the computer-generated predictions was evaluated by calculating the percentage of manually annotated pixels from each feature that were correctly predicted to belong to that category, using the original annotations as a ‘gold standard’ (Fig 6C). Despite the relative simplicity of this approach, we obtained results with reasonable accuracy for many of the annotated features (e.g. median per-pixel accuracy of 68% for sample #1.6.7 in Fig 6). The overall average per-pixel classification accuracy across all histological features from the complete set of 15 annotated tissue sections was 74% (median accuracy of 77%), indicating that considerable predictive success could be obtained despite the fact that these predictive models were based entirely on molecular data without explicitly incorporating morphological or contextual parameters. Notably, repeating the identical classification procedure using only data from the H&E image for sample #1.6.7 (i.e. excluding all other channels from the analysis) led to dramatically worsened performance for the automated feature recognition results, with the median per-feature classification accuracy declining to just 19% (Fig 6D). These findings underscore the critical value of the high-dimensionality in our molecular profiling datasets. Visualizations of the automated histology results for several of these tissue sections are presented as a gallery to illustrate the spatial coherence observed among the predicted tissue features (Fig 7).

To extend the results of our automated histology analysis, we next set out to apply our classification models for computational identification of histological features to independent tissue samples that were not included in the original annotation procedure. We therefore identified independent biological samples included in the TMA dataset that were derived from the same types of normal tissues that were annotated for automated histological analysis. The high degree of anatomical diversity represented among the TMA samples limited the number of independent samples for which we could perform inter-sample feature prediction. However, we were able to carry out inter-sample feature classification on six independent tissue sections from human kidney, breast, liver, and esophagus tissues samples by applying the automated classification models based on the corresponding individual annotated samples of each type (S1 Table). Visualizations of these classification results are depicted in S4 Fig, using the same color-coding scheme for representing features that was applied for the original intra-sample annotations. Although we could not directly evaluate the quantitative accuracy of the automated inter-sample feature predictions for these non-annotated tissue sections, we believe that the spatial coherence of the classification results does illustrate the potential for successful computer-generated *de novo* annotations of histological features.

Finally, we sought to compare the performance of our automated feature classification models with standard methods for histological object identification by performing segmentation of nuclei based on DAPI signal intensities. To identify nuclei by image segmentation we applied a standard algorithm for object recognition using the CellProfiler software package to DAPI images from the 15 samples that were manually annotated (S1 File) [25]. We then constructed annotation-based nuclei images by selecting all pixels from the automated histological predictions that were assigned to features associated with nuclei (S5 Fig). There was strong concordance between the sets of pixels identified as belonging to nuclei from the segmentation and annotation-based analyses with each other, with an average overlap of 84% (median 90%) across all 15 samples (defined as the percentage of pixels in the smaller of the two sets that were classified as nuclei in both sets). Although neither approach appeared to perfectly distinguish all nuclei objects from background across all images, the overall level of agreement between both methods supports the idea that automated classification of relevant histological and subcellular features can be achieved with significant accuracy from multiplex imaging data.

## Discussion

We developed an approach for multi-dimensional microscopic molecular profiling, which relies on iterative cycles of staining and imaging along with a computational pipeline of image processing and analysis scripts for extracting and analyzing the resulting large-scale molecular datasets. The MMMP procedure was compatible with FFPE tissue samples, enabling us to simultaneously analyze over 100 distinct samples in a human tissue microarray. The modularity and flexibility of the overall MMMP approach we employed for this study should enable this analytical strategy to be applied to other contexts as new reagents and technologies available for *in situ* molecular profiling continue to improve and become available. Although the slow cycling times involved in MMMP (limited to 1 cycle per day due to overnight antibody incubations) compare unfavorably to other multiplex imaging technologies [6, 10, 13–15], the ability to implement MMMP without the requirement for specialized equipment or instrumentation may represent an advantage of this approach.

Because our MMMP analysis included histochemical staining of the examined tissue sections, we were able to readily integrate manual annotation of key histological features with our high-dimensional molecular analyses. By doing so, we identified specific molecular profiles associated with annotated features of interest, and we developed automated classification algorithms for recognizing different features based on their underlying molecular attributes. The results obtained from the automated histology classifications were notable, with an overall average per-pixel accuracy of 74% per feature, especially considering that these predictions were based on models that did not explicitly incorporate contextual information related to the morphology or spatial relationships of different features. Thus, we anticipate that predictive models incorporating additional features that are explicitly focused on morphological and spatial context should prove even more successful for automated histology based on high-dimensional profiling data [2, 19]. We expect that further development of computational techniques for automatic feature identification based on multi-parameter molecular imaging data will remain an important area of ongoing investigation, and the results we present may provide a useful framework and resource for advancing future studies. Altogether, these findings contribute to a growing body of evidence that incorporating additional layers of molecular imaging information combined with traditional histochemical images can dramatically enhance the ability to automatically identify histological features with subcellular resolution [10, 14, 15]. The results reported here constitute a step toward development of automated diagnostic systems based on automatic parsing of relevant histological and cellular features from molecular imaging data of arbitrary human tissue samples.

## Materials and Methods

### Immunofluorescence and histochemical staining of TMA slides

Tissue microarrays were derived from anonymized pre-existing samples in the Stanford Pathology Department, and did not involve collection of new tissues for this study. Primary antibodies were obtained from R&D Systems for Lyve-1 (AF2089), CTGF (AF660), SPARC/Osteonectin (AF941), Fzd7 (AFMAB1981), Dkk3 (AF1118), ACE (AF929), Beta-catenin/CTNNB1 (MAB13291), CD105/Endoglin (AF1097), Desmin (AF3844), SMA (MAB1420), from Abcam for CD44 (AB6124) and Collagen I (AB34710), from Bioworld Technology for MMP11 (BS1230) and Col4a2 (BS2399), and from Abnova for CD34 (MAB3835). Purified primary antibodies were directly labeled with Cy5 NHS-ester (Amersham PA3500 or PA35001) following the manufacturer’s instructions. TMA slides with sections of 4 μm thickness were de-paraffinized through three changes of xylene and hydrated to water in 100%, 95%, and 70% ethanol and two changes of de-ionized water. For heat induced epitope retrieval, slides were pre-treated with 1 mM EDTA pH 8.0 at 116°F for 3 minutes using a de-cloaking chamber, then cooled to room temperature (RT). Slides were then blocked with donkey serum at 1:20 dilution in phosphate buffered saline (PBS) for 30 minutes at RT.

The cycle of antibody stain / scan / bleach / scan, was done for each antibody stained on the slide. The slides were incubated overnight for 12–16 hours at 4°C with Cy5 labeled primary antibody. The slides were then washed in PBS and counter-stained with DAPI (ProLong Antifade reagent with DAPI, Invitrogen). Slides were imaged at 20X with a resolution of 0.321 μm per pixel using the Ariol Imaging system v3.2 (Genetix). Slides were then bleached by flushing ozone (Ozone generator A2Z Ozone Inc.) into buffer containing 0.1M citric acid and 0.2M Na_2_HPO_4_ at pH 8 for two hours, then incubated in 10% hydrogen peroxide (Electron Microscopy Sciences) for one hour, and finally incubated in Lugol’s Iodine solution (Electron Microscopy Sciences) for 1 hour. Slides were then washed with de-ionized water and 95% ethanol to remove the Iodine. Following bleaching, the slides were hydrated and then counterstained with DAPI. Slides were imaged to check for any residual Cy5 signal. After all IF antibody stains, slides were stained with a series of histochemical stains (American Master Tech Pentachrome Kit) in the following order: Eosin, Hematoxylin-alcoholic-mayers, Eosin, Alcian Blue, PAS and Verhoeff's Elastic Stain. Slides were imaged and then de-stained after each histochemical stain as follows. Eosin was de-stained with 95% Ethanol for 2–4 minutes; Hematoxylin and Eosin was de-stained with 1% HCl for 10 minutes followed by water for 10 minutes; Periodic acid (which starts the PAS stain) was used to de-stain Alcian blue; and Verhoeff’s elastic stain was applied following PAS without an additional de-staining step.

### Image processing and stitching

Unassembled raw images that tile across each sample were exported directly from the Ariol microscope system. Image metadata was extracted from the associated XML files to relate each set of tiling images to its original sample within the TMA, using a customized PERL script <File-S1-parse-Ariol-XML.pl>. All brightfield images were mathematically negated to ensure compatibility with subsequent image stitching and registration algorithms. For each imaging cycle, the tiling images spanning each tissue sample were then automatically assembled together using a FIJI macro for image stitching [23] [22]. Image stitching was performed independently three times using different values for the preferred degree of overlap (Input parameter “overlap” = 5, 8, or 10), and the parameter setting which agreed best with the consensus (based on Euclidian distance of the inferred x and y offset values for each tile) was then used to generate the final single stitched image for each tissue section. The resulting stitched images for each cycle were then cropped to a region with dimensions 2300 by 2300 pixels. In order to correctly track the stitched image files between cycles, centroids of the (x,y) coordinates associated with each core on the TMA were computed for every cycle, and the centroids of all samples on the TMA were globally aligned across cycles using the custom R script <File-S2-grid-Align.R>.

### Registration of images in the MMMP series

Full image alignments for each tissue section were generated according to a multi-step procedure. First, grayscale images containing normalized intensities derived only from the DAPI-channel (blue) from each immunofluorescence (IF) cycle were sequentially registered with each other based on rigid transformations using the FIJI “Register Virtual Stack Slices” macro [23]. The transformation parameters underlying the multiple alignment of IF images based on their DAPI signals were saved and then applied to the original (non-grayscale) IF images. Then, bright-field images from all histochemical staining cycles were negated and registered with each other using all color channels (red, green, and blue) and the same FIJI macro. Next, each of the IF images were aligned to the first IF image from the series based on their DAPI intensities using a FIJI registration algorithm allowing elastic transformations [21]. Finally, the first brightfield image from H&E staining was rigidly aligned to the last IF image using the FIJI “Register Virtual Stack Slices” macro, and the saved transformations were then appropriately applied to all of the images in the MMMP series to produce the final sequence of registered images. For these steps, version 1.46j of Fiji / ImageJ and Java 1.6.0_65 (64-bit) were used on the Mac operating system version 10.

### MMMP data extraction and unsupervised analysis

Signal intensities were extracted by converting each registered image from the MMMP series into text format using ImageMagick software and parsing out the values associated with every pixel for each relevant image channel. Further analysis was restricted to “foreground” regions that were identified as being present in all aligned images from the MMMP series. For each sample, a MMMP data matrix of molecular profiles was constructed with each row corresponding to a foreground pixel within the tissue section, and 29 columns corresponding to the different image channels extracted from the MMMP series (2 DAPI channels from the first and last IF images, 15 Cy5 channels from each of the IF images, and 12 columns derived from the red, green, and blue channels from each of the 4 histochemical images). Principal components analysis (PCA) was performed on the scaled MMMP data using the R statistical analysis software (version 1.40) (www.r-project.org). Clustering analysis was also carried out in R using the “kmeans” function with the following parameter settings: centers = 100, nstart = 1, iter.max = 20, algorithm = “H”. Similarity-based color coding of the *k*-means clusters was performed by multi-dimensional scaling of the cluster centroids into three-dimensional space and transformation of the resulting (x,y,z) coordinates into RGB color values. Full details are available in the attached R script <File-S3-MMMP-analyze.R>.

### Analysis of annotated histological features in tissue sections

Relevant cellular and histological features of interest within select tissue sections were manually annotated using the registered H&E image from the respective MMMP series as a reference. The coordinates of image pixels annotated as belonging to each category of features were extracted, and each labeled feature was designated an arbitrary color code for visualization purposes. Molecular profiles associated with each annotated feature were identified by performing *k*-means clustering as before (*k* = 100) separately on the MMMP data vectors derived from each histological category. The pairwise Euclidian distances were then calculated for all of the resulting cluster centroids associated with all histological features, and clusters were determined to be feature-specific if their nearest neighbor belonged to the same annotated feature as they did. Full details are available in script <File-S4-MMMP-classify.R>.

### Automated classification of histological features

Each annotated image was conceptually partitioned into two discrete regions (Region 1 and Region 2) based on a “checkerboard” pattern consisting of an alternating grid of square segments. The grid size (i.e. the edge length for alternating squares) was set as the maximum value of 100, 50, or 20 pixels for which both partitions included one or more pixels belonging to every annotated category. The MMMP data vectors associated with Region 1 were used as a “training set” to build a multi-label classifier based on linear discriminant analysis (LDA), which was then applied to data from Region 2 as an independent “test set” to generate automatically predicted feature annotations based on the underlying molecular profiles. Then, the reciprocal analysis and classification was performed using Region 2 as the “training set” and Region 1 as the “test set”. The automated classifications resulting from both analyses were then merged to create a color-coded output image representing the de novo prediction of annotated features within the tissue section. Classification accuracy of the automated prediction was assessed by calculating the percentage of manually annotated pixels from each feature that were correctly predicted to belong to that category. Full details are available in script <File-S4-MMMP-classify.R>.

## Supporting Information

S1 Fig Example of Image Stitching Artifact Correction. Image stitching results for the first MMMP cycle of Lyve1-antibody staining of human colon tissue sample (#1.6.7) are shown as an example. Images were generated using either the original default software provided with the Ariol microscope (a,c) or using the computational image processing pipeline we developed (b,d). Close-up views of the same corresponding tissue region indicated by black squares in the full-size images are shown at 6X magnification for the original (c) and corrected (d) versions of the stitched image.
(TIF)

S2 Fig Comparison of MMMP Results and Replicate Antibody Staining for Ace. (A) MMMP images showing staining for Angiotensin I converting enzyme (Ace) using Cy5-labeled primary antibody (red) during the fourth cycle of the MMMP series are shown for samples of normal human endometrium (#1.6.6), fallopian tube (#1.9.6) and kidney (#2.7.9) tissue sections. (B) Replicate staining for Ace was performed independently on separate tissue sections obtained from the same samples. All of the sections were co-stained with DAPI to visualize nuclei (blue).
(TIF)

S3 Fig *k*-means Analyses of MMMP Data with Varying Cluster Size Parameters. (a) Molecular profiling data from a human small bowel terminal ileum tissue section (#1.10.7) was clustered using a *k*-means algorithm with different values for the cluster size parameter *k* set to either *k* = 10 (A), 100 (B), or 1000 (C). The centroid vectors obtained from each of the clustering results were compiled together and used to generate a single similarity-based color-mapping transformation based on multi-dimensional scaling. Visualization of the cluster membership for each pixel was performed as in Fig 3, and the centroid vectors from each analysis were themselves clustered and displayed in the heatmaps below each visualization.
(TIF)

S4 Fig Automated Feature Prediction Applied to Independent Tissue Samples. Original feature prediction results based on internal partitioning and classification of annotated tissue sections are shown for normal human breast (#1.3.1), liver (#1.8.10) and kidney (#2.7.9) samples (top row). Computational models for histological feature classification from each of these samples were then applied to generate automated predictions for independent breast (#1.6.1), liver (#.1.7.10) and kidney (#2.5.8) samples of the corresponding type, which are visualized using the same color-coding scheme as for the original annotations.
(TIF)

S5 Fig Comparison of Nuclei Identification from Image Segmentation and Annotation-Based Feature Classification. Nuclei were identified from MMMP images for 15 tissue sections using two independent methods. Conventional image segmentation of nuclei objects based on DAPI signal intensity was performed. Separately, annotation-based nuclei identification was performed by merging all nuclei-associated classifications generated by the histological feature predictions. For each sample, the pixels identified as belonging to nuclei according to both methods are shown in blue, with those identified exclusively using one approach are shown in magenta for segmentation-based identification and in cyan for annotation-based identification.
(TIF)

S1 File Archive of Supporting Information Files. Includes: (File A) PERL Script for Processing Image Coordinates, (File B) R Script for Tracking Image Location Coordinates Between Cycles, (File C) R Script for Unsupervised Analysis of MMMP Data, (File D) R Script for Analysis & Classification of Annotated Histological Features, (File E) CellProfiler Pipeline for Nuclei Segmentation.
(ZIP)

S1 Table Description of Tissue Samples Contained on TMA. (XLS)

S2 Table Principal Component Analysis Summary Data for All Samples. (XLS)

S3 Table K-means Cluster Analysis Summary Data for All Samples. (XLS)

S4 Table Summary Data for Feature-Specific Molecular Profiles. (XLS)

S5 Table K-means Centroids Identified with Different Cluster Size Parameters. (XLS)
