# Supplementary figures and images for: Automated Analysis and Classification of Histological Tissue Features by Multi-Dimensional Microscopic Molecular Profiling
Source: PLoS One. 2015 Jul 15;10(7):e0128975. doi: 10.1371/journal.pone.0128975 (PMC4503351; doi:10.1371/journal.pone.0128975)

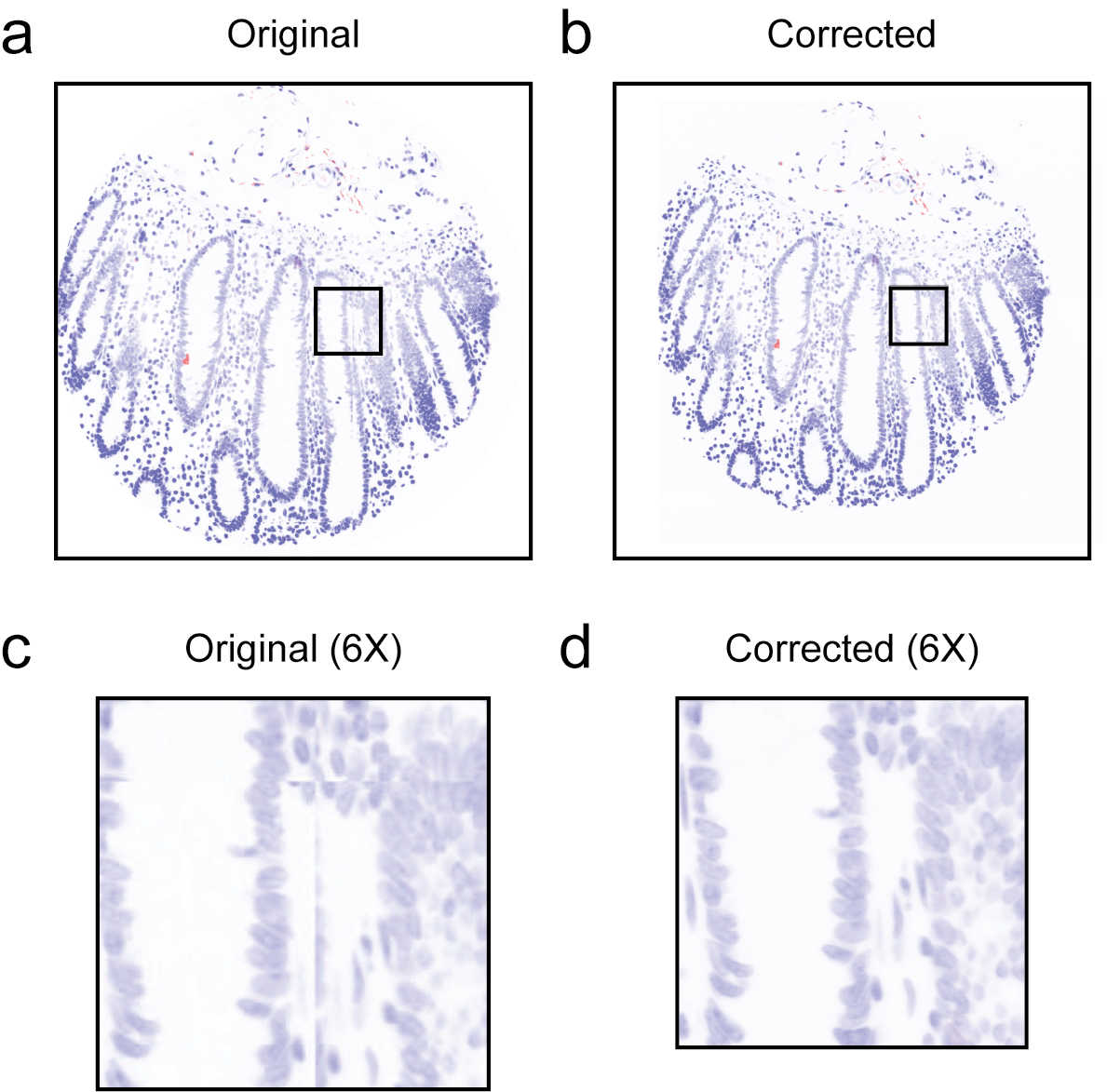

Supplement: S1 Fig — Image stitching results for the first MMMP cycle of Lyve1-antibody staining of human colon tissue sample (#1.6.7) are shown as an example. Images were generated using either the original default software provided with the Ariol microscope (a,c) or using the computational image processing pipeline we developed (b,d). Close-up views of the same corresponding tissue region indicated by black squares in the full-size images are shown at 6X magnification for the original (c) and corrected (d) versions of the stitched image. (TIF) [file pone.0128975.s001.tif]

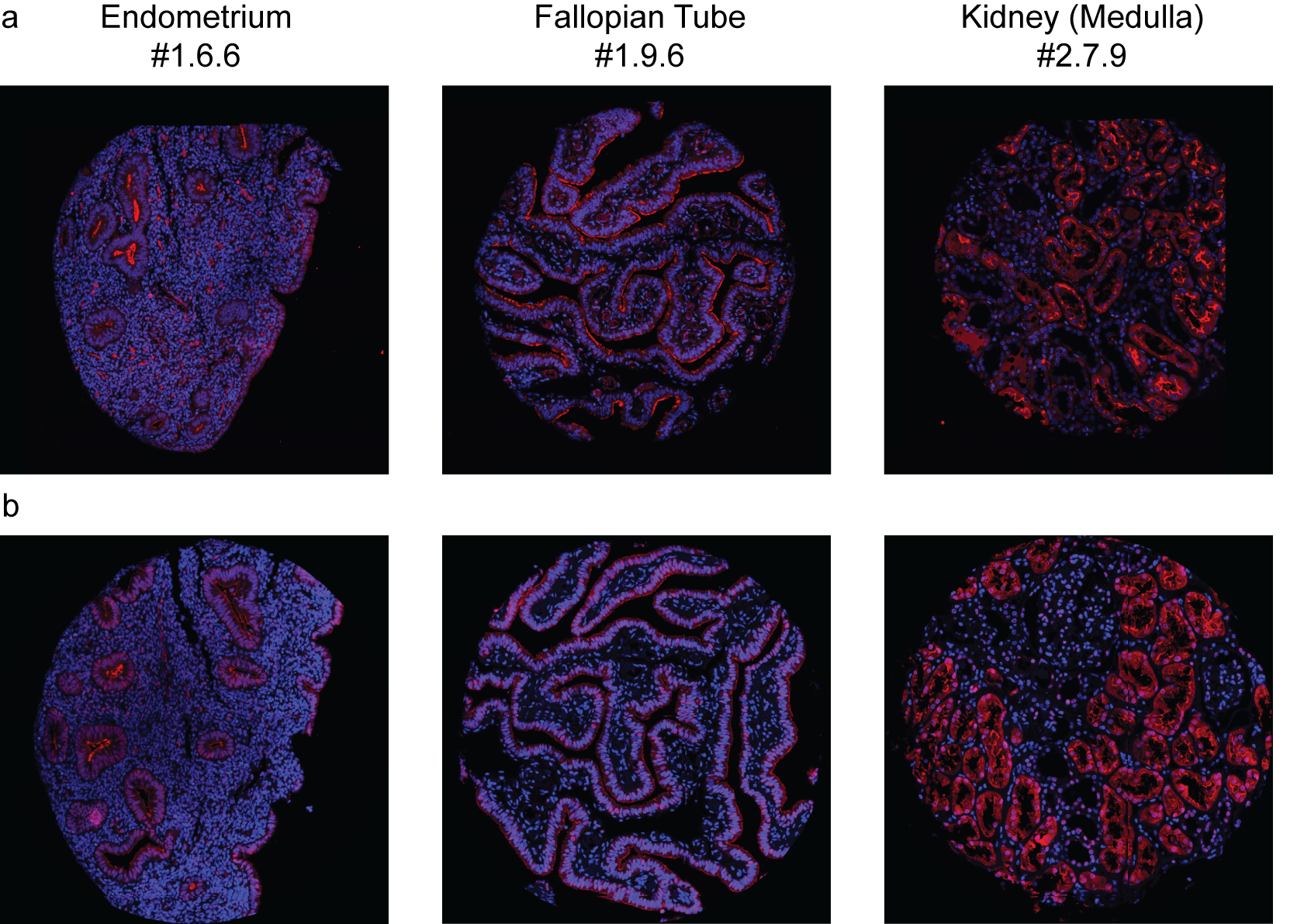

Supplement: S2 Fig — (A) MMMP images showing staining for Angiotensin I converting enzyme (Ace) using Cy5-labeled primary antibody (red) during the fourth cycle of the MMMP series are shown for samples of normal human endometrium (#1.6.6), fallopian tube (#1.9.6) and kidney (#2.7.9) tissue sections. (B) Replicate staining for Ace was performed independently on separate tissue sections obtained from the same samples. All of the sections were co-stained with DAPI to visualize nuclei (blue). (TIF) [file pone.0128975.s002.tif]

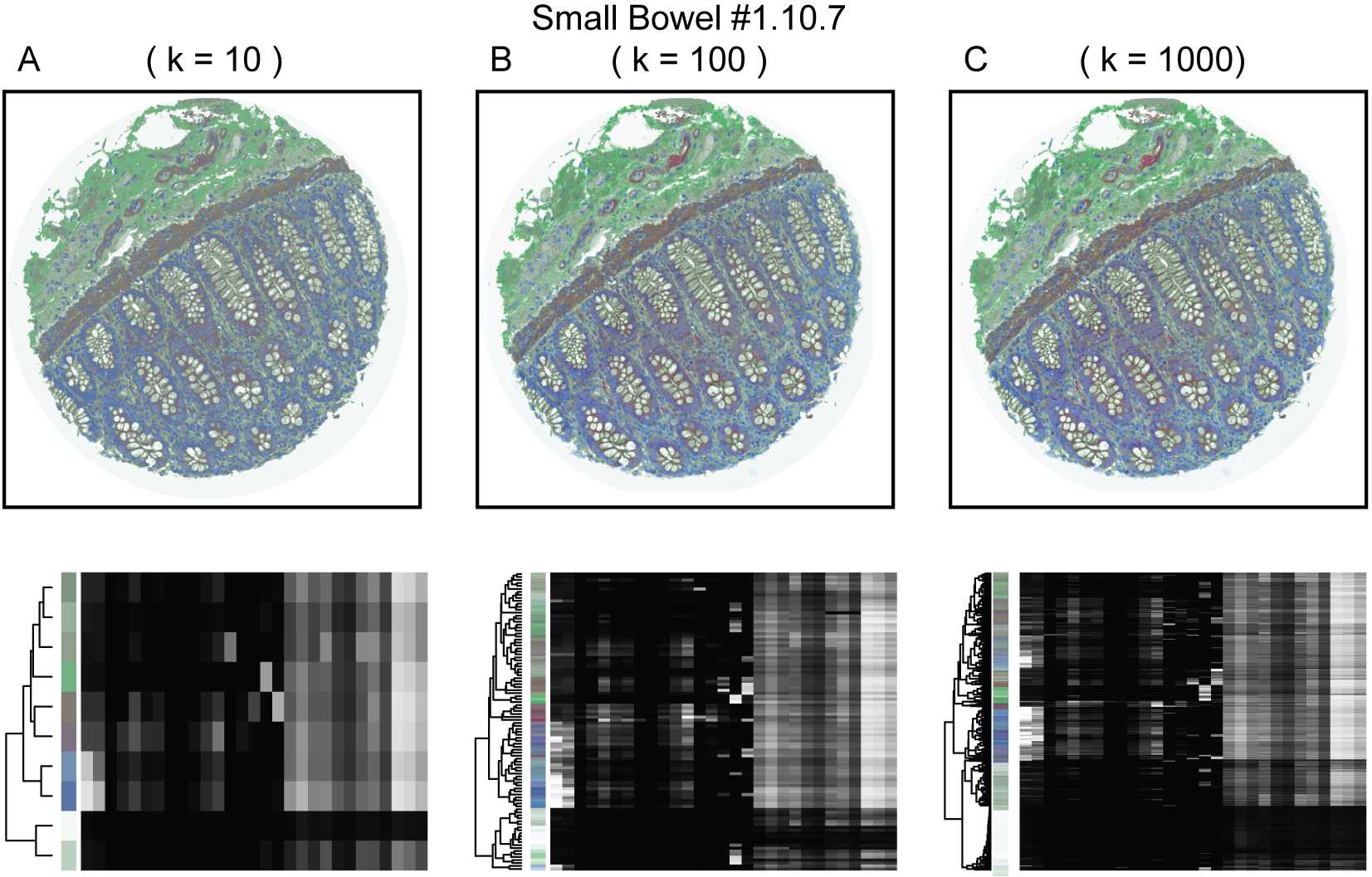

Supplement: S3 Fig — (a) Molecular profiling data from a human small bowel terminal ileum tissue section (#1.10.7) was clustered using a k-means algorithm with different values for the cluster size parameter k set to either k = 10 (A), 100 (B), or 1000 (C). The centroid vectors obtained from each of the clustering results were compiled together and used to generate a single similarity-based color-mapping transformation based on multi-dimensional scaling. Visualization of the cluster membership for each pixel was performed as in Fig 3, and the centroid vectors from each analysis were themselves clustered and displayed in the heatmaps below each visualization. (TIF) [file pone.0128975.s003.tif]

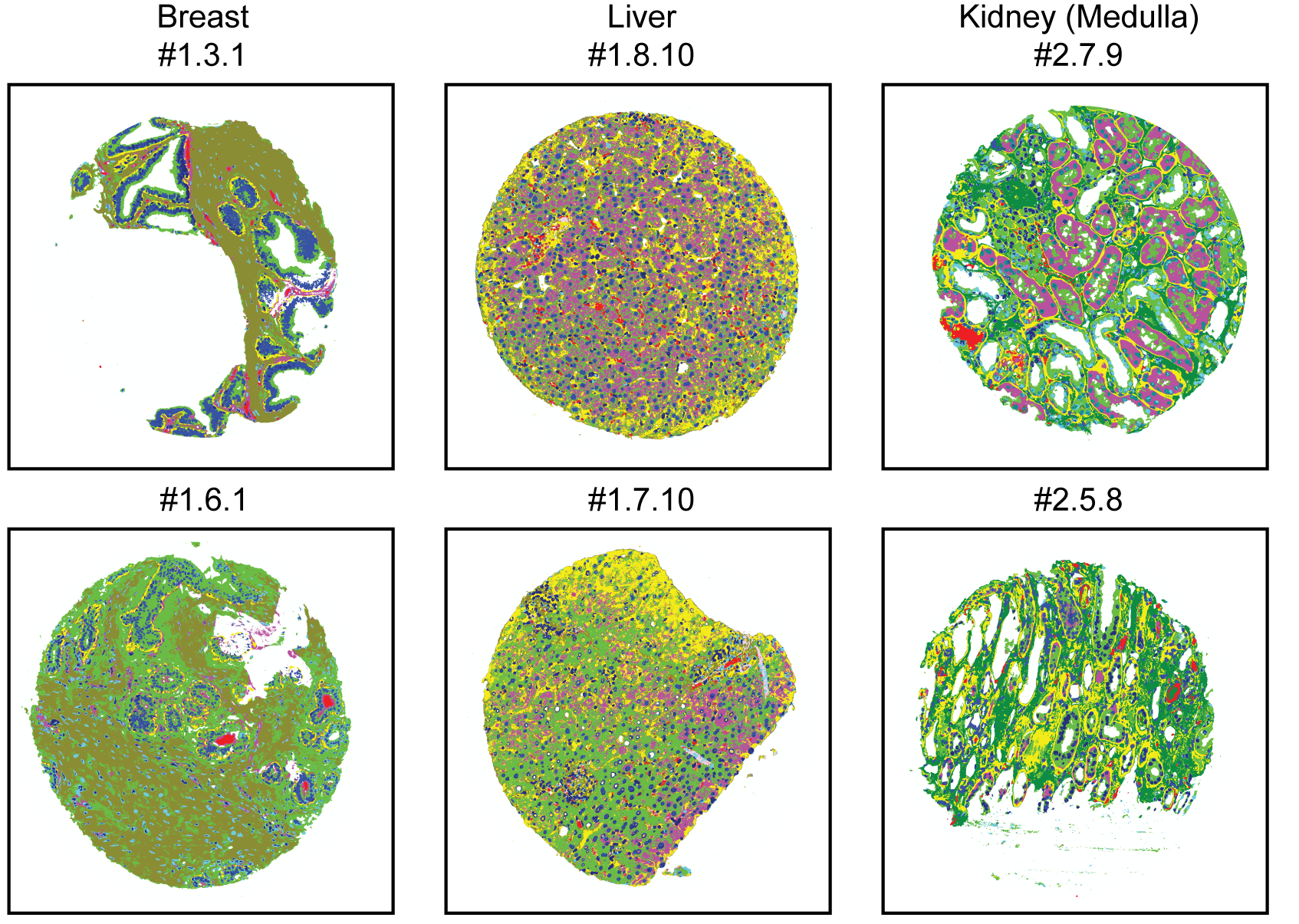

Supplement: S4 Fig — Original feature prediction results based on internal partitioning and classification of annotated tissue sections are shown for normal human breast (#1.3.1), liver (#1.8.10) and kidney (#2.7.9) samples (top row). Computational models for histological feature classification from each of these samples were then applied to generate automated predictions for independent breast (#1.6.1), liver (#.1.7.10) and kidney (#2.5.8) samples of the corresponding type, which are visualized using the same color-coding scheme as for the original annotations. (TIF) [file pone.0128975.s004.tif]

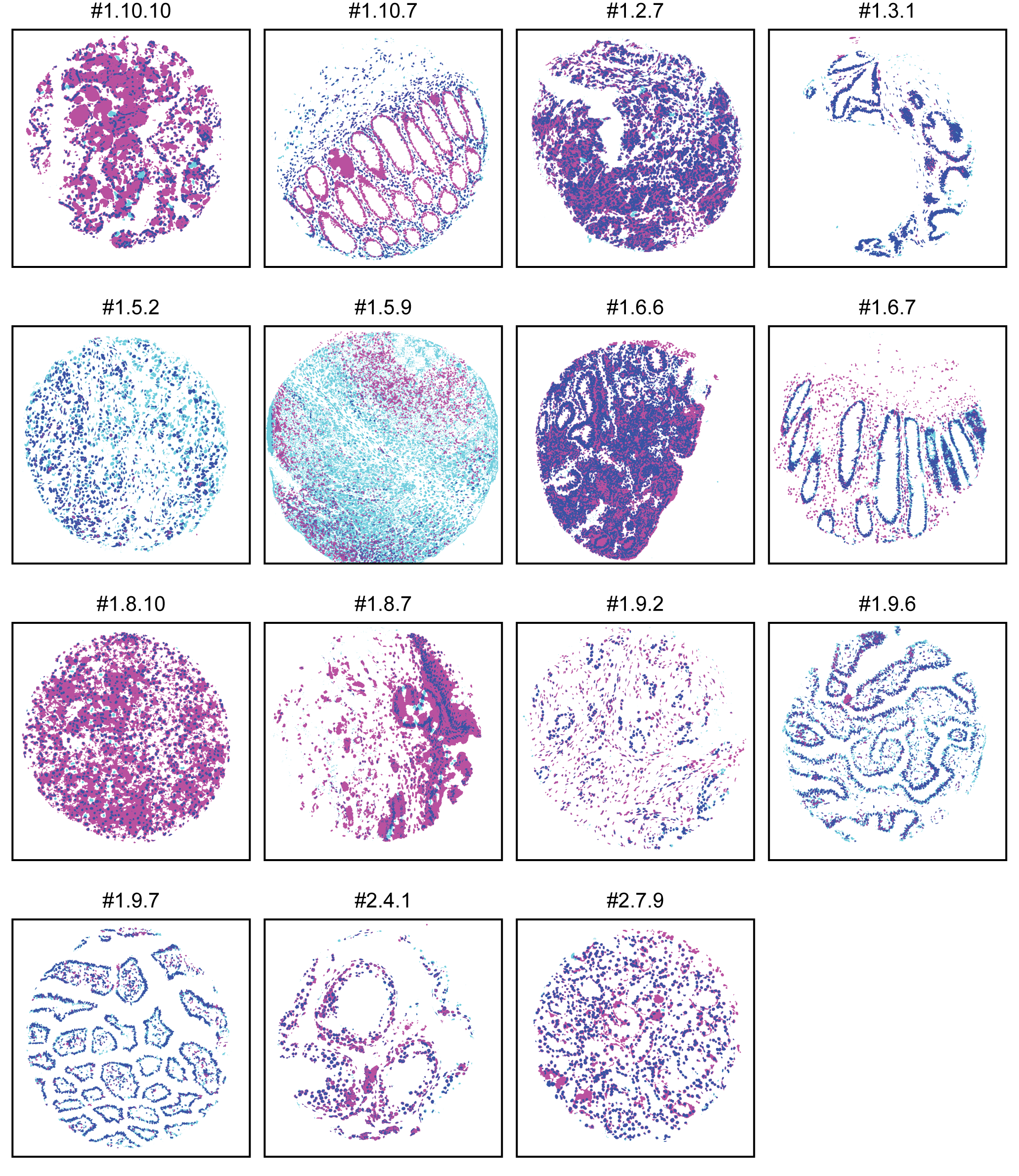

Supplement: S5 Fig — Nuclei were identified from MMMP images for 15 tissue sections using two independent methods. Conventional image segmentation of nuclei objects based on DAPI signal intensity was performed. Separately, annotation-based nuclei identification was performed by merging all nuclei-associated classifications generated by the histological feature predictions. For each sample, the pixels identified as belonging to nuclei according to both methods are shown in blue, with those identified exclusively using one approach are shown in magenta for segmentation-based identification and in cyan for annotation-based identification. (TIF) [file pone.0128975.s005.tif]
